# Supplementary material for: Polymer Patterning by Laser-Induced Multipoint Initiation of Frontal Polymerization
Source: ACS Appl Mater Interfaces. 2024 Feb 28;16(14):17973–80. doi: 10.1021/acsami.4c00216 (PMC11009908; doi:10.1021/acsami.4c00216)
Supplement: Supplementary file 1 — am4c00216_si_001.pdf [file am4c00216_si_001.pdf]

SUPPORTING INFORMATION:

Polymer Patterning by Laser-Induced Multipoint Initiation of Frontal Polymerization

Andrés L. Cook[1], Mason A. Dearborn[2], Trevor M. Anderberg[1], Kavya Vaidya[2], Justin E. Jureller[3], Aaron P. Esser-Kahn[2,4], Allison H. Squires[2,4*]*

[1]Department of Physics, University of Chicago, Chicago, IL, 60637 USA
[2]Pritzker School of Molecular Engineering, University of Chicago, Chicago, IL, 60637 USA
[3]James Franck Institute, University of Chicago, Chicago, IL, 60637 USA
[4]Institute for Biophysical Dynamics, University of Chicago, Chicago, IL, 60637 USA
* E-mail correspondence: aesserkahn@uchicago.edu and asquires@uchicago.edu

Contents

SUPPORTING NOTES: 3

SI NOTE 1: INITIATION TIME CALCULATION 3

SI NOTE 2: SYNCHRONICITY OF INITIATION 4

SI NOTE 3: PATTERN INVERTIBILITY 5

SUPPORTING FIGURES: 6

Figure S1: Example of initiation time calculation for seven FP samples 6

Figure S2: Camera images of three multipoint samples 7

Figure S3: Temperature vs time plot at the initiation points of a four-point sample 8

Figure S4: Height profile of a line cut across a seam 8

Figure S5: Seam invertibility. 9

SUPPORTING NOTES:

SI NOTE 1: INITIATION TIME CALCULATION

In order to determine the time to initiation, we measured the temperature of individual initiation points. The temperature data initially follows an exponential curve consistent with a Newtonian heating profile. We define the time of divergence from this profile as the initiation time. To quantify this, we select a linear region of the Newtonian curve, fit a line to that portion of the temperature profile, and determine the first point at which the data is at least 10% higher than the linear prediction. The resulting annotated temperature profiles are shown in Figure S1. The three vertical lines show the start and end of the fitting window (black), as well as the laser activation time (red), set as the common "zero time" for all profiles. The purple line is the linear fit, and the red X marks the initiation time.

The resulting power vs. time graph follows a reciprocal trend. This suggests a fixed energy cost to initiate polymerization:

$$t = \frac{\Delta E_{\text{thresh}}}{P} \tag{S1}$$

In Eqn. S1, ΔE_{thresh} is the polymerization energy, t is the initiation time, and P is the initiation power. We determine ΔE_{thresh} by fitting Equation S1 to our experimental data, as shown in Figure **2a**. Because transport effects increase the variation in initiation time for large t, we weight our fit by the inverse of the initiation time. This fit results in a polymerization energy of approximately 300 mJ (294 ± 108 mJ).

SI NOTE 2: SYNCHRONICITY OF INITIATION

Shown in Figure S3 is a set of temperature traces for a four-point sample that displayed unusual lack of synchronicity, likely due to poor mixing or the presence of large clusters of carbon black particles. This asynchronicity allows us to explore the factors limiting the efficacy of multipoint initiation. The divergence of our measured temperature traces from a Newtonian heating profile does not seem to occur at a fixed initiation temperature, but rather spontaneously at some point along the profile. This apparent stochasticity of the temperature divergence limits the simultaneity of polymerization initiation, and therefore the spatial precision of seam placement, despite the laser's spatial precision in delivering energy.

The traces of Figure S3 display a second unusual feature also found in other temperature profiles: a "shoulder" occurring between 50 and 100 degrees Celsius. In this case, the shoulder lasts an average of 1.25 s, while the fronts from that sample travel at an average speed of 1.82 mm s^{-1}. Therefore, the front travels 2.28 mm during the shoulder, approximately equal to the 2.5 mm sample depth, suggesting that the shoulder may be caused by the fronts (which initiate at the top of the monomer due to carbon black's high absorption) traveling to the bottom of the dish as a hemispherical wave before transitioning to a cylindrical wave in the bulk monomer.

The degree of synchronicity of polymerization initiation directly dictates the design tolerances that are achievable for any desired pattern of seams. Because our fronts travel with a speed, v_f, on the order of mm s^{-1} and have a width of order 1 mm (see Figure S4), the error in front displacement, Δx, should be of the same order of magnitude as the front width if the difference in initiation time between the two points, Δt, is on the order of a few seconds:

$$\Delta t \approx \frac{\Delta x}{v_f} \tag{S2}$$

To test our resolution in seam placement, we measured the distance from a seam to its initiation points (along the line connecting the two initiation points) in a four-point sample. We subtracted half the inter-point distance to obtain the seam positioning error, here measured to be 0.04 ± 0.12 mm (mean ± standard deviation). On these grounds, we describe our initiation as synchronous.

SI NOTE 3: PATTERN INVERTIBILITY

While we can generate initiation patterns for many desired seam patterns, not every seam pattern will produce a unique initiation pattern; (1) some seam patterns can be created by infinitely many initiation patterns, (2) some can be created by exactly one, and (3) some are not invertible at all. Figure S5 shows examples of (1) and (3). The seam pattern shown in Figure S5a has a continuum of possible initiation patterns: any rescaled version of the triangular pattern shown in blue will lead to the same seam pattern. By contrast, the seam pattern in Figure S5b has no inverse solutions. This is because any pair of initiation points that form a seam are reflections of each other about the axis of the seam. In order to form the 180° angle shown in the pattern, the leftmost initiation point must be a reflection of both points on the right, which is geometrically impossible on a planar sample. For more discussion of the mathematical underpinnings of inverse seam prediction, please see Ref. 34.

SUPPORTING FIGURES:

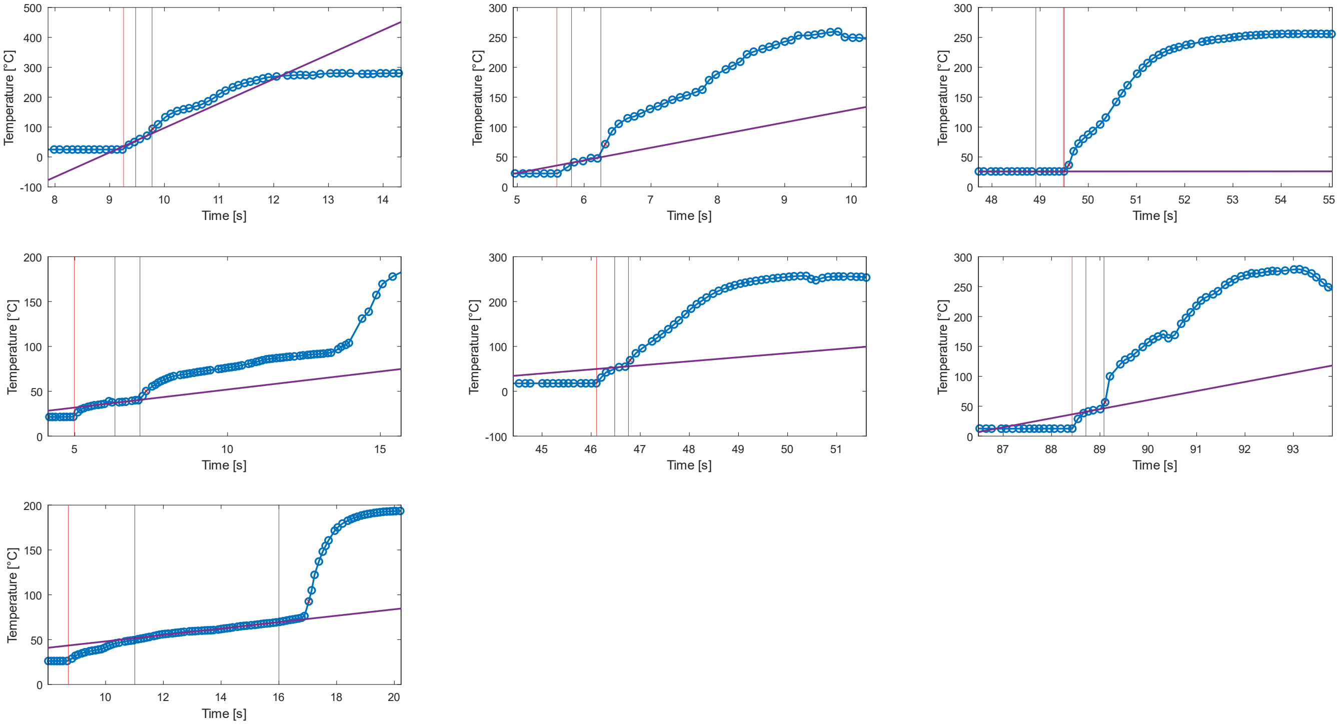

Figure S1: Example of initiation time calculation for seven FP samples, showing guidelines used to designate the fitting window (black) and laser activation time (red). The initiation time calculation algorithm produces a linear fit (purple) and initiation time (red x)

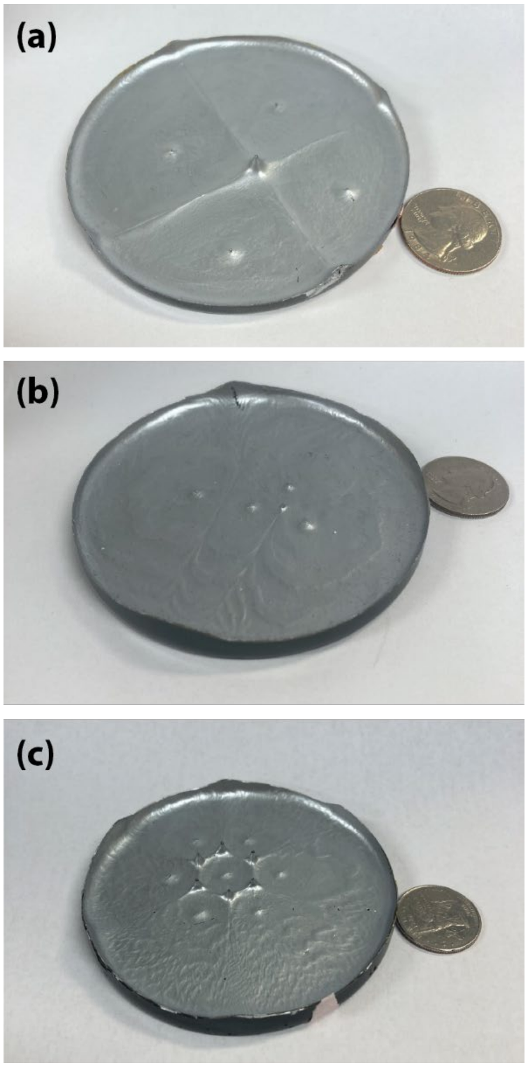

Figure S2: Camera images of three multipoint samples, showing seams between initiation points, peaks where three or more seams meet, and shallow bumps at the initiation points. Samples were painted silver to improve image contrast and photographed using a smartphone camera with a quarter dollar coin (USA) for scale. **(a)** Four points, square pattern. (**b)** Four points, "T" pattern. **(c)** Seven points.

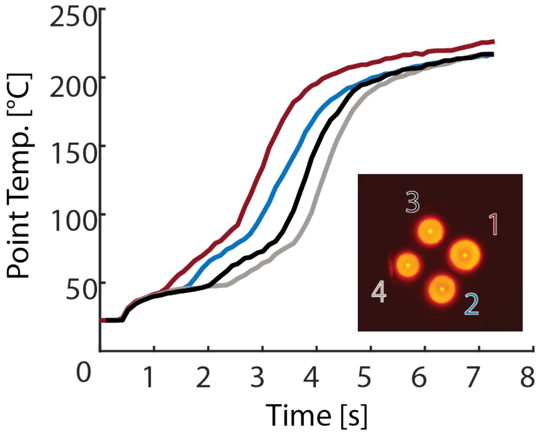

Figure S3: Temperature vs time plot at the initiation points of a four-point sample. Inset: Thermal images with initiation points color-coded corresponding with the appropriate temperature curves.

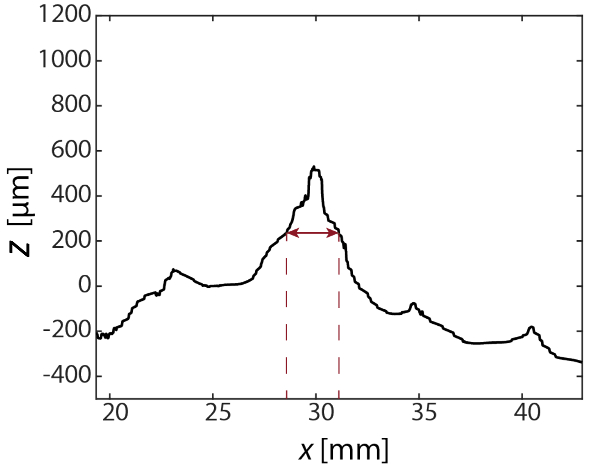

Figure S4: Height profile of a line cut across a seam demonstrating a seam width on the order of ~1 mm. *x* and *z* are measured with respect to an arbitrary origin.

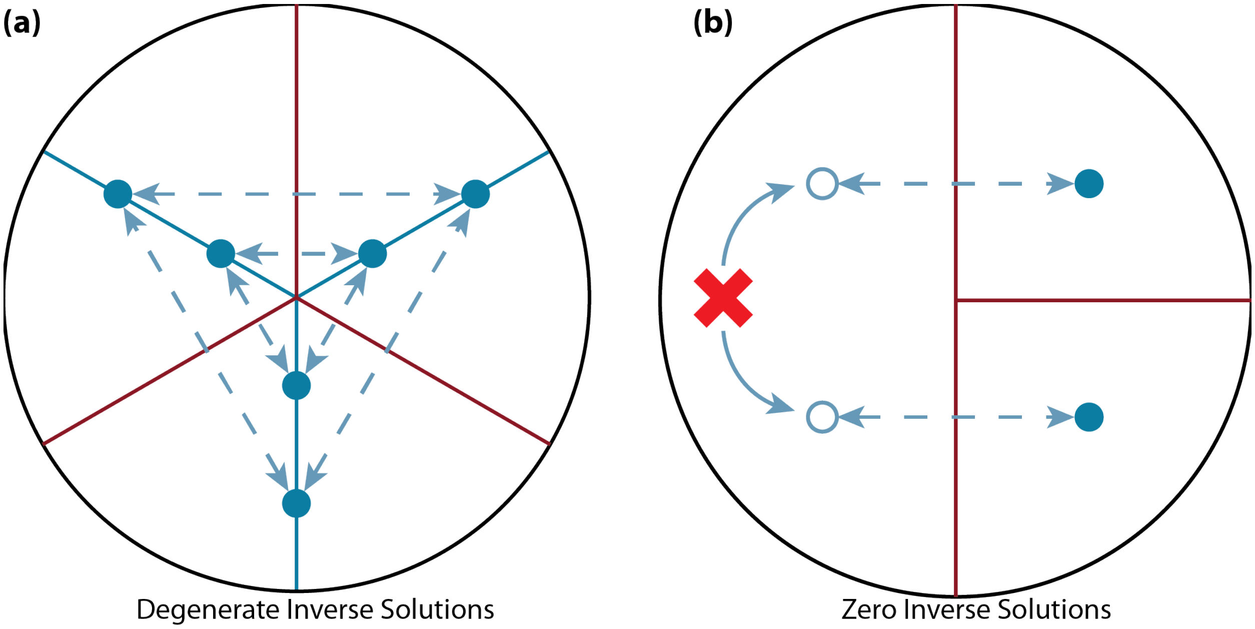

Figure S5: Seam invertibility. **(a)** A pattern with infinitely many inverses. Red lines show desired seams, while blue lines show possible initiation sites. Two possible sets of initiation sites, with seam normals, are shown in blue. **(b)** A pattern with no inverse. Again, red lines show desired seams and blue dots show initiation points. The required points to form the two “halves” of the vertical seam are shown as empty blue circles and are incompatible.
